# SigSel: A MATLAB package for the pre and post-treatment of high-resolution mass spectrometry signals using the ROIMCR methodology

**DOI:** 10.1016/j.mex.2023.102199

**Published:** 2023-04-25

**Authors:** Carlos Pérez-López, Antoni Ginebreda, Damia Barcelo, Roma Tauler

**Affiliations:** a Institute of Environmental Assessment and Water Research (IDAEA-CSIC), Department of Environmental Chemistry, Jordi Girona 18–26, Barcelona 08034, Spain; b Catalan Institute for Water Research (ICRA-CERCA), Emili Grahit 101, Parc Científic i Tecnològic de la Universitat de Girona, Edifici H2O, Girona 17003, Spain

**Keywords:** Chemical compound identification, Quantitative analysis, Mass spectrometry, Metabolomics, Non-target analysis, SigSel

## Abstract

The Regions of Interest Multivariate curve Resolution (ROIMCR) methodology has gained significance for analyzing mass spectrometry data. The new SigSel package improves the ROIMCR methodology by providing a filtering step to reduce computational costs and to identify chemical compounds giving low-intensity signals. SigSel allows the visualization and assessment of ROIMCR results and filters out components resolved as interferences and background noise. This improves the analysis of complex mixtures and facilitates the identification of chemical compounds for statistical or chemometrics analysis.

SigSel has been tested using metabolomics samples of mussels exposed to the sulfamethoxazole antibiotic. It begins by analyzing the data according to their charge state, eliminating signals considered background noise, and reducing the size of the datasets. In the ROIMCR analysis, the resolution of 30 ROIMCR components was achieved. After evaluating these components, 24 were ultimately selected explaining 99.05% of the total data variance. From ROIMCR results, chemical annotation is performed using different methods:

• Generating a list of signals and reanalyzing them in a data-dependent analysis.
• Comparing the ROIMCR resolved mass spectra to those stored in online repositories.
• Searching MS signals of chemical compounds in the ROIMCR resolved spectra profiles.

## Graphical abstract

**Figure:**
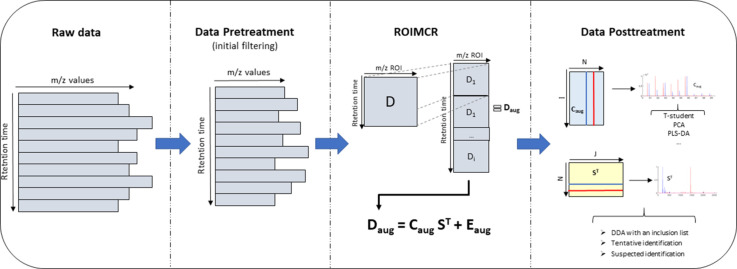

Specifications table

**Table:** 

Subject area:	Bioinformatics
More specific subject area:	Data management
Name of your method:	SigSel
Name and reference of original method:	n.a.
Resource availability:	https://github.com/cplqam/SigSel.git

Method details

## Introduction

In recent years, Liquid Chromatography-High Resolution Mass Spectrometry (LC-HRMS) has become a widely used method in analytical studies [1]. Despite the wealth of information obtained from LC-HRMS analysis that allows for the complete characterization of samples, the storage and computational management of the large amounts of data generated can still present challenges. To address these storage and computational tasks, various computer software and algorithms have been developed. Some of these are commercial, with their workflows typically hidden from the user, while others are open-source, such as MS-Dial [2], XCMS [3,4], MaxQuant [5] or Regions of Interest- Multivariate Curve Resolution-Alternating Least Squares (ROIMCR) [6,7].

The ROIMCR methodology is composed of two coupled techniques. The first one involves filtering and compressing the mass spectrometry data using the concept of Regions of Interest (ROI) [8]. The second technique is the application of the bilinear factor decomposition of the filtered Regions of Interest (ROI) data matrix to determine the concentrations and spectral profiles of the components in the analyzed mixture using the Multivariate Curve Resolution-Alternating Least Squares (MCR-ALS) method [9]. Initially developed for the non-target analysis of complex mixtures in proteomics [10], metabolomics [11,12] and lipidomics [13,14] studies, the ROIMCR methodology has more recently been applied to environmental studies [15,16]. Despite the success of the ROI method in compressing and filtering mass spectrometry data, some ROI signals may still be associated with background noise and not be fully filtered. To address this issue, the Signal Selection (SigSel) package, a MATLAB tool, is presented. It allows for the exploration of raw high-resolution mass spectrometry data signals and the management of signals with undefined charges, which can be considered noise. The package enables the user to delete these uncharged noisy signals if necessary, optimizing the first ROI step. Additionally, SigSel provides various post-processing tools to extract information from the MCR-ALS results, making it easier to annotate and identify the chemical constituents present in the analyzed samples, and facilitating their quantitative analysis. In this work, the SigSel method workflow and details are described, and its application is illustrated using an example from a metabolomics study.

## Workflow

The workflow of SigSel for the analysis of mass spectrometry datasets in the original instrumental acquisition raw format is composed of 3 steps (detailed below): a) Data transfer, visualization, and filtering based on the charge of the measured MS signals, b) data compression and filtering using the Regions of Interest (ROI) method and Multivariate Curve Resolution (MCR) componentization (ROIMCR method) and c) Extraction of chemical information from ROIMCR results to carry out the annotation and identification of the resolved components from their elution and spectra profiles, as well as the quantitative assessment of the observed concentration changes of these components among the analyzed samples and their posterior statistical, chemometrics and pattern recognition analysis. All the developed functions used in this work are in MATLAB programing language, and they are available in the GitHub repository https://github.com/cplqam/SigSel.git.

### Charge exploration and filtration

The raw MS datasets contain both the desired chemical signals and those from the instrumental background, noise, and solvent interferences. In these cases, low-intensity signals of sought-after chemical compounds can sometimes be removed from the data due to the use of a too-high ROI noise threshold. To prevent this loss of chemical information, an additional pre-filtering step based on the charge of the signals can be applied to reduce the ROI intensity threshold applied afterward and achieve an increase in the sensitivity of the whole analysis.

**Fig. 1:**
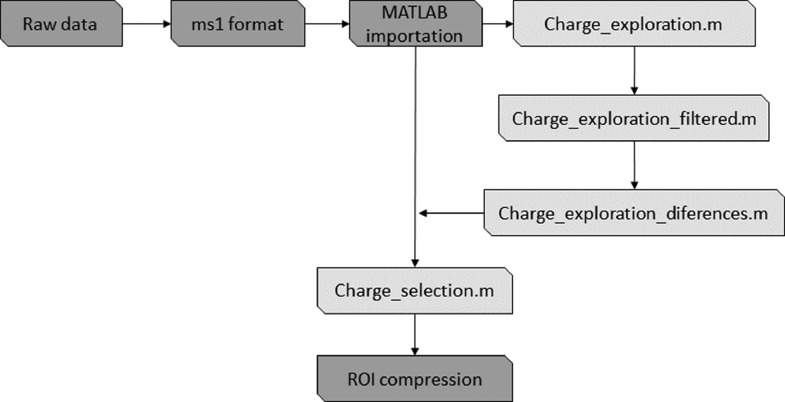
Workflow *for* charge exploration and filtering before ROIMCR analysis.

The proposed SigSel workflow for selecting mass spectrometry signals based on their charge is illustrated in Fig. 1 and explained in more detail in the Pre_ROIMCR file (Supplementary information). Using a format transformation program to obtain MS1 format (see in the next section), signals with charge 0 (or not defined), charge 1, or a charge greater than 1 can be distinguished from the raw data format originally acquired by the mass spectrometry instrument (Thermo instruments). After analyzing the charge distribution, the option to remove signals with specific charges is provided. This results in a significant reduction of the total number of MS signals recorded, allowing for a reduction of the ROI signal intensity threshold and reducing computational storage requirements without losing relevant information. The ROI procedure can be applied to the charge-filtered MS signals either directly by using the MATLAB command line program environment [7] or by using the Mass Spectrometry Regions of Interest Graphical User Interface (MSroi GUI) tool [17]. In the latter case, the original raw datasets are imported in the mzXML format in MSroi GUI, and the generated variables are replaced with those from the results obtained from the SigSel pretreatment process.

### The ROIMCR methodology

Once the charge-filtered datasets are in the MATLAB computing environment, they are compressed and analyzed using the ROIMCR methodology (http://www.mcrals.info), which has been described in more detail in other works [7,10,13]. Briefly, ROIMCR is divided into 2 steps, a first step of signal filtering and compression using the ROI concept [8], and a second step that analyzes the ROI data matrix using the MCR-ALS method [6,18,19]. Application of the ROI compression approach gives a data matrix where only those MS signal regions reliable and relevant are considered. The selection of these MSROIs is performed depending on three parameters: the background noise MS signal intensity threshold, the MS instrument mass accuracy and the minimum number of m/z signals that define an elution peak at the chromatographic resolution conditions. Once the MSROI data matrix is built, its bilinear factor decomposition by the MCR-ALS method [6,18,19] gives two-factor matrices related respectively with the mass spectra and elution profiles of the different constituents of the sample and therefore with their qualitative (identification) and relative quantitative information. When more than one sample is analyzed simultaneously, the quantitative information provided by the ROIMCR can be used to analyze the changes in the concentrations of the chemical compounds among samples or to apply other statistical and pattern recognition chemometrics analyses such as Principal Component Analysis (PCA) [20], Partial Least Squares-Discriminant Analysis (PLS-DA) [21,22] or ANOVA Simultaneous Component Analysis (ASCA) [22]. For more specific details, see the references previously given for both the ROI compression and MCR-ALS analysis.

### Identification and quantification of chemical compounds from ROIMCR results

The SigSel package can be used to evaluate the results of the ROIMCR. Initially, the MCR components are analyzed, their profiles are examined (visually), and those that can be associated with noise signals rather than chemical compounds (the shapes of the elution and spectra profiles that are unfeasible from a chemical perspective) can be eliminated if desired. Signals from components associated with background noise can be removed before further processing.

**Fig. 2:**
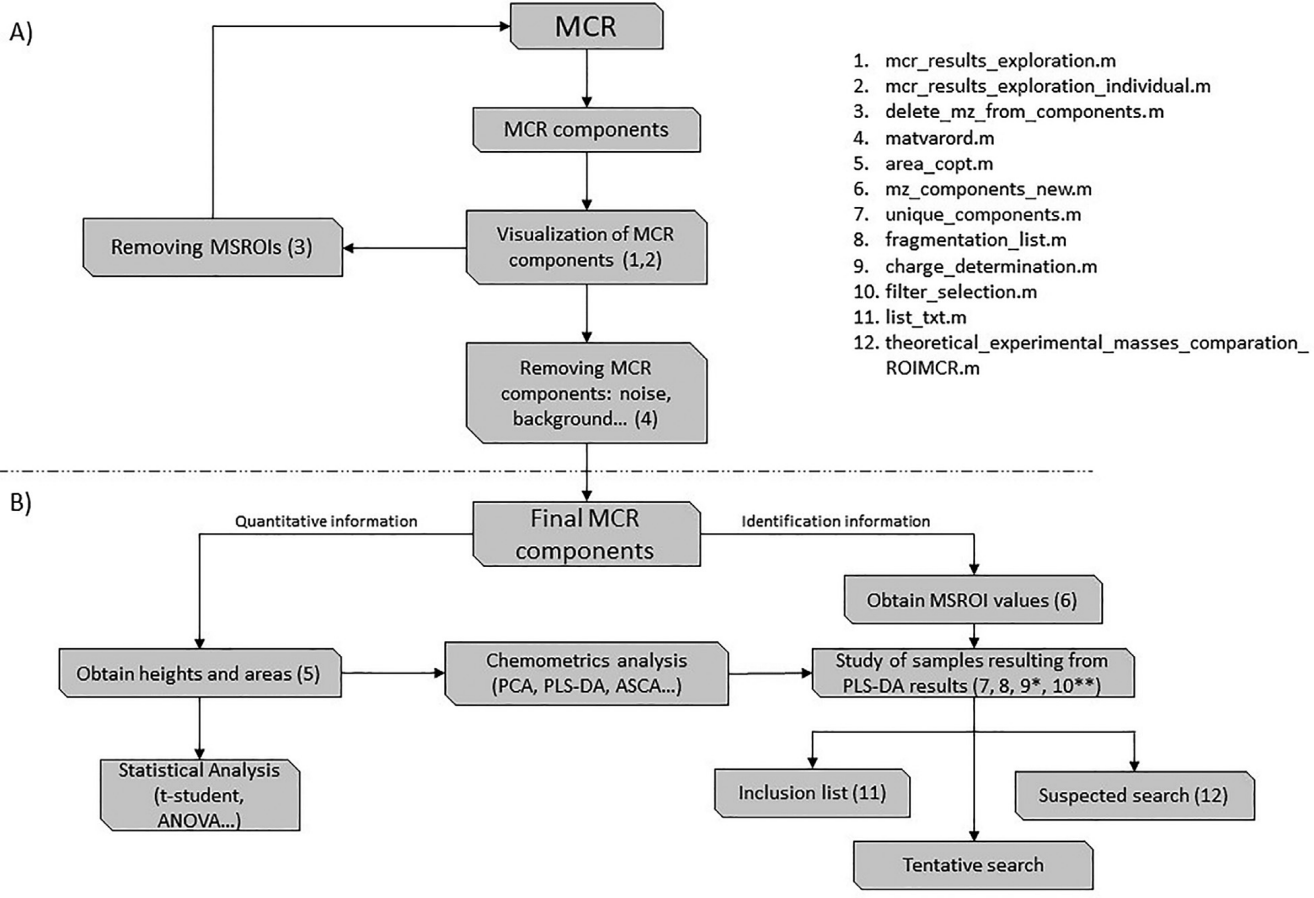
Workflow used for the interpretation of ROIMCR results. In parentheses, the identification number of the function in SigSel package is given. In part A) new SigSel tools for the visualization and selection of MCR components, and in part, B) new SigSel tools for the analysis of ROIMCR results. *Only when a list of possible suspected compounds is included to be reanalyzed in a DDA analysis. **Only when the identification of MCR components is performed using chemometrics and statistical analyses.

After this initial quality control and selection of the MCR resolved components, the peak areas and heights of their elution profiles can be obtained and submitted to further statistical and chemometric analysis to study the concentration variations of the different chemical compounds among samples. Additionally, the mass spectra of each MCR resolved component can be used to annotate and identify the associated chemical compounds using different strategies to facilitate the chemical compound identification: generating a list of MSROI signals and reanalyzing them in a Data Dependent Analysis (DDA) to determine their fragmentation patterns, comparing the ROIMCR resolved mass spectra to those stored in online repositories and public databases, and searching MS signals of known chemical compounds in the ROIMCR resolved elution and spectra profiles (Fig. 2).

In summary, SigSel is a useful tool for evaluating and interpreting the results obtained from the ROIMCR method when analyzing mass spectrometry datasets from multiple samples. It allows for more efficient and organized data analysis, leading to reliable conclusions. The workflow of the various options for visualization, evaluation, and data extraction is illustrated in Fig. 2 and is also described in the Post_ROIMCR file (Supplementary information).

## Case of study

The capabilities of the SigSel software package can be better understood by using an experimental data example from a previous LC-MS metabolomics study of mussel (*Mytilus galloprovincialis*) samples exposed to the antibiotic sulfamethoxazole in two different seasons. The samples were analyzed by LC-HRMS using an LC-LTQ Orbitrap Velos™ spectrometer. Further information can be found in previous work [23]. The 3 steps of the SigSel method previously mentioned in workflow section were applied to process the LC-HRMS data acquired in the analysis of 4 of these samples (2 control and 2 exposed to sulfamethoxazole). Although the metabolomics datasets of this work are used as an example, the same workflow has options for its adaptation for proteomics studies as it is explained in the following section. The raw files of these 4 samples are available in a folder in the GitHub repository https://github.com/cplqam/SigSel.git.

### Charge exploration and filtration

Despite using four samples in this example, only one of them will be analyzed and discussed in detail in this section, following the workflow shown in Fig. 1, for the sake of simplicity. However, all samples will be studied, filtered, and used in the next steps. Firstly, RawConverter software [24] was first applied to the instrument raw datasets to convert them to MS1 signals, including their charge information [21]. After this initial data transformation, MS1 signals were imported directly to the MATLAB environment as a numeric data matrix after replacing the missing values with 0. The resulting imported sample is stored in a single-column cell array, where every cell has the MS ROI data matrix of one sample, allowing for sequential analysis of more than one sample in the case of a multisample study.

**Fig. 3:**
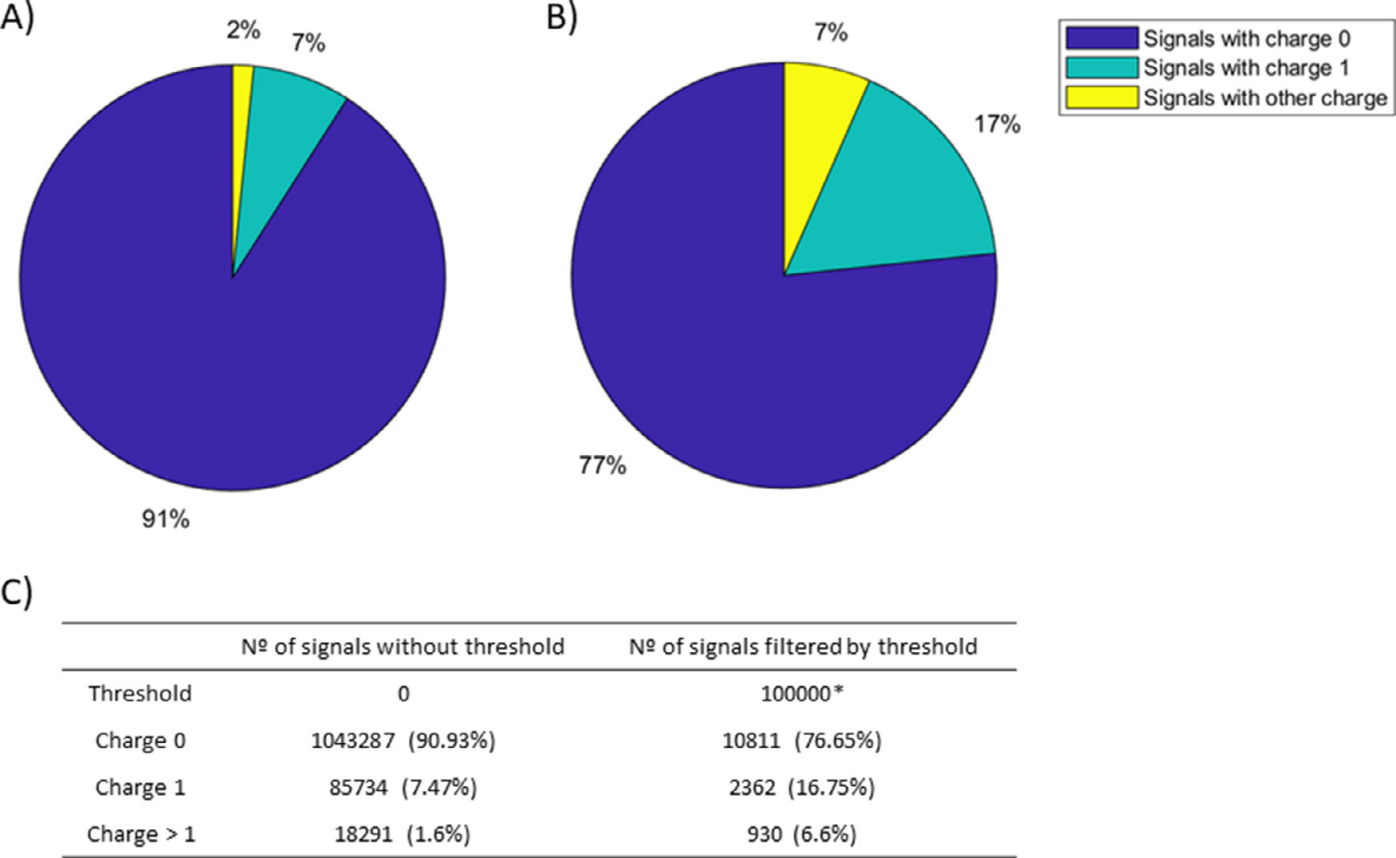
Number and percentage of MS signals filtered by the SigSel charge exploration workflow in the analysis of a metabolomics sample. A) Percentage of signals depending on their charge state (0, 1 or >1), B) Percentage of signals with an intensity higher than the selected threshold and C) Numerical data values of A and B Fig.s. *The chosen intensity threshold has been 0.5% of the maximum MS signal intensity.

As seen in Fig. 3A of this metabolomics sample analysis, a significant number of mass spectrometry (MS) signals were identified as having a charge state of 0 or with a not defined charge by the instrument. As shown in Figs. 3B and 3C, many of these undefined charge signals have low-intensity levels and are similar to background noise. Therefore, they can be classified as background or interference signals and can be removed. This removal results in a significant reduction in file size, improving computer storage and processing time for subsequent ROIMCR analysis. Removing signals with an undefined charge has the benefit of allowing for a lower threshold intensity to be applied during the subsequent ROI filtering process. These MS signals with a charge state of 0 (or with no defined charge) are removed from the raw data files before they are analyzed by ROIMCR [17]. This increases the number of lower-intensity MS signals with charge state 1 or higher that can be correctly attributed to specific chemical compounds. The option of removing signals with charge 1 can be particularly useful in proteomics analyses [25], as it enables the removal of non-peptide signals generated by trypsin fragmentation.

The MS signals were filtered by their charge state before being analyzed using the ROI procedure. An intensity threshold of 25,000 u.a was used, which was significantly lower than the original threshold of 50,000 u.a. This was possible due to the removal of signals with charge state 0 or not defined. The mass error tolerance was set to 0.005 Da and a minimum of 6 signals were required to define a chromatographic peak.

### ROIMCR analysis

The ROI procedure was applied to all 4 metabolomics samples simultaneously, resulting in a column-wise augmented data matrix with 290 columns, corresponding to the number of MSROI signals selected in the analysis of all 4 samples. The number of rows in this matrix was equal to the total number of MS scans (retention times) in the LC analysis of all samples. This method of data matrix augmentation is particularly useful for comparing trends among samples and for subsequent processing (as described below).

MCR-ALS model with 30 components was able to explain 99.33% of the total data variance. Increasing the number of components did not reveal any additional information about the chemical constituents in the analyzed samples since they would be related to background noise and explain low data variance. Some of the 30 MCR components identified were not actual chemical constituents of the samples but were related to MS signals from the solvent, background, and instrumental noise [11]. Further evaluation and filtering of these non-sample related MCR components were performed using various visualization and filtering options in SigSel (as described in the next section)

### Interpretation of ROIMCR results

The interpretation of the ROIMCR results is a two-step process: first, visualization and quality control of the MCR components, and second, extraction of qualitative and quantitative information to identify the chemical compounds associated with the MCR components and determine their relative concentrations in the analyzed samples. This information is then used for statistical and chemometrics analyses. Fig. 2 provides a detailed illustration of the different steps and functions of the SigSel package used to interpret ROIMCR results.

#### Visualization and quality control of MCR results

**Fig. 4:**
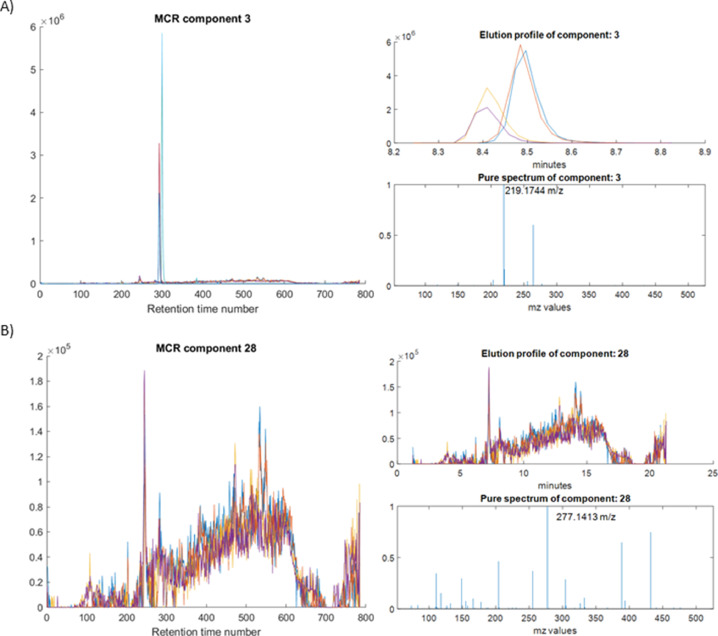
Elution and spectra profiles of two components resolved by ROIMCR in the analysis of the 4 metabolomic samples visualized using the SigSel package. In the two Figs. A and B, on the left the elution profiles in the four metabolomic samples are plotted against the retention time number, on the right top, the elution profiles in the four metabolomic samples are plotted against the retention times in minutes (zoomed) and on the right bottom, the resolved mass spectrum of the same component is plotter against the m/z v¡alues. In Fig. A) the resolved MCR profiles are associated with a metabolite of the analyzed sample, and in B) the resolved MCR profiles are associated with background MS signals.

Following the workflow shown in Fig. 2A, the 30 MCR components obtained from the ROIMCR analysis are visualized and evaluated. As an example, Fig. 4 illustrates two different MCR components, one associated with a chemical compound (Fig. 4A) and the other with noise, background, or instrumental signals (Fig. 4B). In this case, the component shown in Fig. 4B may be removed from the MCR model. Additionally, if a single MCR component is found to explain a large percentage of the total variance, potentially obscuring the presence of other minor but significant components, the MSROI signals from its spectrum can be removed from the data matrix and the MCR analysis can be repeated.

By following this process, 24 MCR components were ultimately selected out of the initial 30, as they are likely associated with the chemical compounds (metabolites) present in the analyzed samples. These components explained 99.05% of the total data variance. The elution and spectra profiles associated with these MCR components can be utilized for identification, as well as statistical and chemometrics analyses.

#### Identification of the possible chemical compounds present in the unknown sample and statistical and chemometrics analyses of the observed changes of their concentrations in different samples simultaneously analyzed

The peak heights and areas of the elution profiles of the MCR-resolved components were calculated as shown in Fig. 2B. This information was then utilized in statistical or chemometric multivariate analyses, such as PCA, PLS-DA, or ASCA, to investigate the chemical compounds (metabolites) responsible for differentiation among groups of samples, and to determine if this variation was statistically significant (e.g. using Wilcoxon or t-student tests). To further interpret the biological and environmental significance of the obtained results, annotation and identification of the MCR-resolved components are necessary. This can be achieved by using both in-house and publicly accessible databases and repositories.

**Fig. 5:**
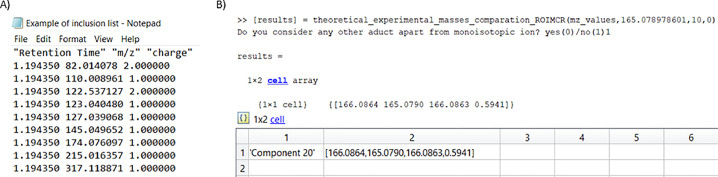
Example of SigSel options for chemical compound identification. A) a list with the retention times, m/z values and their charge state to DDA MS/MS new reanalysis of the samples; B) searching what MCR component is associated with a suspected compound (phenylalanine) using its molecular adduct m/z, theoretical monoisotopic molecular weight, ROIMCR m/z value and deviation in ppm.

In this example, those MCR components confirmed to be reliable were identified and checked for their presence in the different analyzed samples and examined in more detail using some optional steps. The m/z values associated with each resolved mass spectrum were used for chemical identification using three different methods. The first method uses a list of retention times, MS1 m/z values, and charge state information from SigSel results to obtain their corresponding MS2 spectra from a posterior DDA analysis (Fig. 5A). The second method uses a list of results from SigSel to search in a web repository or database, such as Human Metabolome Database (HMDB) [26] or MassBank [27] for tentative identification of the chemical compound. The third method uses a suspected list of possible sample compounds with known mass spectra information to search for a match with the MS signals resolved for the different MCR components. In Fig. 5B, Phenylalanine was suspected to be present in the samples and the associated MCR component was found by comparing the monoisotopic molecular weight value (obtained from HMDB) of this chemical compound with the ROIMCR m/z values and giving the error in ppm.

## Conclusions

ROIMCR methodology has been proposed in omics and environmental studies [[10], [11], [12], [13],^15^] to obtain comprehensive and reliable information about the presence of chemical compounds in complex environmental and biological sample mixtures analyzed by HRMS. In order to facilitate the extraction of the information resulting from ROIMCR results and to identify the chemical compounds present in the analyzed samples, as well as to allow their posterior quantitative, statistical and chemometrics analyses, the SigSel package presented in this work provides a simple workflow to achieve these tasks. The familiarization and implementation of SigSel can result in an easy way to obtain valuable information from the ROIMCR results, by avoiding the manual inspection of every single compound one by one, which can result in a laborious and time-consuming task. As shown in the example provided in this work, filtering raw MS datasets by their charge reduces significantly the computer storage and allows better management of the MS datasets and a better resolution of chemical compounds giving low intensity MS signals (e.g. because they are at very low concentrations), without increasing the computational cost. On the other hand, the visualization tools of the MCR results provided by SigSel facilitate the possibility of eliminating those MCR components related to background and noisy signals, improving the reliability of the results and their posterior statistical and chemometric analysis. The important identification step, essential to environmental or biological interpretation, is also simplified by SigSel, since the discovery of the m/z signals belonging to the sought chemical compounds is easier, including suspected and tentative identifications, as well as the necessary information to be introduced in the MS instrument in case of posterior confirmatory MS2 analysis of the sample were needed.

## Ethics statements

N.A.

## CRediT authorship contribution statement

**Carlos Pérez-López:** Conceptualization, Methodology, Software, Writing – original draft. **Antoni Ginebreda:** Data curation, Writing – review & editing. **Damia Barcelo:** Supervision, Project administration. **Roma Tauler:** Data curation, Writing – review & editing.

## Declaration of Competing Interests

The authors declare that they have no known competing financial interests or personal relationships that could have appeared to influence the work reported in this paper.

## Data Availability

I have shared the link to my data and code in the main manuscript.

## Appendix Supplementary materials

Image, application 1

Image, application 2
