# Supplementary material for: SigSel: A MATLAB package for the pre and post-treatment of high-resolution mass spectrometry signals using the ROIMCR methodology
Source: MethodsX. 2023 Apr 25;10:102199. doi: 10.1016/j.mex.2023.102199 (PMC10326443; doi:10.1016/j.mex.2023.102199)
Supplement: Supplementary file 1 [file mmc1.docx]

**Pseudocode of postreatment SigSel functions**

Pseudocode of the SigSel functions for post-ROIMCR workflow (Figure 2 in main text). Here the functions are explained sequentially, following the numeric order given in Figure 2. Black letter lines have the name of the function, the input and the output variables, and green letter lines have the pseudocode of each SigSel function. For more information about these variables see the SigSel functions in GitHub repository (link in main text).

mcr_results_exploration(copt,sopt,mz,nsam,time)

%Obtain the number of rows of c matrix

%For each sample

%Obtain the retention times

%end

%Repeat this process with the rows of the c matrix to store its values in diferent

%rows in cell array

%For each component in s matrix

%For each sample

%plot the elution profile from matrix of each sample overlapped

%end

%If zoom is required for elution profile

%Ask the user for the interval to zoom in and ensure an interval is introduced

%Create a subplot

%In a first plot the zoomed elution profile from matrix of each

% sample overlapped

%In a second plot create a barplot with the spectrum information

%Save graph and pause for user visualization

%Elseif zoom is not required for elution profile

%Create a subplot

%In a first plot the zoomed elution profile from matrix of each

% sample overlapped

%In a second plot create a barplot with the spectrum information

%Save graph and pause for user visualization

%end

%Clear the plots

%end

%end

mcr_results_exploration_individual(copt,sopt,mz,nsam,time,comp)

%Obtain the number of rows of c matrix

%For each sample

%Obtain the retention times

%end

%Repeat this process with the rows of the c matrix to store its values in diferent

%rows in cell array

%For each sample

%plot the elution profile from matrix of each sample overlapped

%end

%Ask for zoom in the times where the chemical compound is eluting

%Repeat question til user say 1 or 0 (yes/no)

%If zoom is required for elution profile

%Ask the user for the interval to zoom in and ensure an interval is introduced

%Create a subplot

%In a first plot the zoomed elution profile from matrix of each

% sample overlapped

%In a second plot create a barplot with the spectrum information

%Save graph and pause for user visualization

%Elseif zoom is not required for elution profile

%Create a subplot

%In a first plot the zoomed elution profile from matrix of each

% sample overlapped

%In a second plot create a barplot with the spectrum information

%Save graph and pause for user visualization

%end

%Clear the plots

%end

[MSroi_new,mzroi_new] = delete_mz_from_components(d,mz,s,perc)

%Ask for the MCR component user wants to delete and ensure it exists

%Create a variable like the results from mz_components_new.m

%Select those components choosen by the user to be deleted

%For each component selected to be deleted

%Store in an array variable the m/z values

%end

%For each m/z value previously stoed

%Find its position in MSroi and mzroi matrix

%end

%Delete the columns with these positions in MSroi and mzroi matrixes

[dred,mzred,colelim,cred,sred,cnew,snew,r2new,r2old]=matvarord(d,c,s,time,mz)

%Ask for ploting reordered profiles and spectra

%Ask for inspecting or eliminating in the reordered data matrix

%Ask for intensity threshold

%Obtain the variance of each MCR component and the total variance

%Sort the components depending on their explained variance

%For each component

%If display is true

%Plot the elution profile of the MCR component in a subplot and

% the spectrum of the MCR component in a subplot

%end

%Calculate the threshold

%If inspection opion has been chosen

%Ask for elimination the MCR component and eliminate in the case

%user select it

%end

%end

%Display the columns eliminated

%Display the mz eliminated

%Display the MCR components eliminated

%Generate the filtered variables as output

%end

[area,height] = area_copt(copt,nsamp,nrows)

%Obtain the dimensions of the c matrix

%For each sample

%Obtain the rows in c matrix

%For each component

%Obtein the area and maximum height

%end

%end

%Plot the area and height of all components

%end

[resultados] = mz_components_new(mz,s,perc)

%Obtain the rows of s

%For each row

%Obtain the maximum value

%For each column

%store the position of the value iif it is higher than threshold

%end

%Obtain the m/z values from position data

%Obtain the component numbers

%end

[result1,result2] = unique_components(copt, times,per)

%Ask for number of samples

%Obtain the number of columns of copt

%Obtain the max value for each column

%For each sample

%Separate the rows of copt that belong to this sample

%end

%For each column

%Obtain the threshold

%For each sample

%If maximum is higher than threshold, add the sample

%end

%If only 1 sample is in the list of representation for 1 MCR component

%This component is included in a list of only 1 sample representation

%end

%end

%Sort the outputs

%end

[resultado1, resultado2] = fragmentation_list(copt,times,signals,mz,per,m_time)

%Ask for the number of samples

%Obtain the retaention times and rows of copt matrix for each sample

%Obtain the number of columns of signals

%For each column

%Obtain the samples represented in this MCR component

%For each sample represented in this MCr component

%Obtain the threshold and store the signals higher than this one

%and their retention times

%end

%Store the results

%end

%Organize the stored results

%For each sample

%Associate the mz of each component and the retention times of that component

%end

%end

res_final = charge_determination(file,signals,error)

%If signals with not defined charge have been eliminated

%Ask for the elimination of signals with charge 1

%For each sample

%Clare the signals seleccted to be eliminated and obtain the charge

%of the rest signals

%end

%elseif signals with not defined charge have not been eliminated

%For each sample obtain the charge of signals from file variable

%end

%For each sample

%Obtain the signals, charges and rows of the sample

%For each row of the sample

%Obtain the m/z value and interval

%For each row in charge file

%Store it if it is inside the interval

%end

%end

%Store the results

%end

%end

[resultado] = filter_selection(file,samples,components)

%Obtain the samples of interest

%For each sample of interest

%For each MCR component of interest

%Select the signals belong to these components for each sample

%end

%end

%end

list_txt(lista)

%Obtain the number of samples

%For each sample

%At each column print the title

%For each row

%Print the m/z, intensity and charge values in the txt file

%end

%end

%end

[resultado] = torical_experimental_masses_comparation_ROIMCR(file,mz_teo,ppm_permitido, ionization)

%Obtain the number of components

%If ionization is equal to 0

%If user wants to include other aducts rather than monoisotopic ion

%Ask for these aducts and calculate molecular mass

%For each MCR component

%Search if some m/z has an error lower than ppm_permitido

%Store in a variable for more possible results

%end

%elseif no

%Calculate molecular mass

%For each MCR component

%Search if some m/z has an error lower than ppm_permitido

%Store in a variable for more possible results

%end

%Store in a variable for more possible results

%end

%elseif ionization is equal to 1

%Ask for other aducts rather than monoisotopic ion

%If user wants to include other aducts rather than monoisotopic ion

%Ask for these aducts and calculate molecular mass

%For each MCR component

%Search if some m/z has an error lower than ppm_permitido

%Store in a variable for more possible results

%end

%elseif no

%Calculate molecular mass

%For each MCR component

%Search if some m/z has an error lower than ppm_permitido

%Store in a variable for more possible results

%end

%Store in a variable for more possible results

%end

%end

%end
