# Supplementary material for: SigSel: A MATLAB package for the pre and post-treatment of high-resolution mass spectrometry signals using the ROIMCR methodology
Source: MethodsX. 2023 Apr 25;10:102199. doi: 10.1016/j.mex.2023.102199 (PMC10326443; doi:10.1016/j.mex.2023.102199)
Supplement: Supplementary file 2 [file mmc2.docx]

**Pseudocode of pretreatment SigSel functions**

Pseudocode of the SigSel functions for pre-ROIMCR workflow (Figure 1 in main text). Here the functions are explained sequentially, following the order given in Figure 1. Black letter lines have the name of the function, the input and the output variables, and green letter lines have the pseudocode of each SigSel function. For more information about these variables see the SigSel functions in GitHub repository (link in main text).

[result, result_c0,result_c1,result_cresto] = charge_exploration(file)

%Import samples following the instructions provided in function input description

%For each sample

%Obtain number of rows

%For each row

%Obtain the scan number and retention time

%Add these values in fifth and sixth respectively

%end

%Find the rows with no signal information and delete them

%Count the signals with charge 0 and display

%Count the signals with charge 1 and display

%Count the signals with other charges and display

%Show a piechar if user select

%end

[result, result_c0,result_c1,result_cresto] = charge_exploration_filtered(file)

%Import samples following the instructions provided in function input description

%For each sample

%Obtain number of rows

%For each row

%Obtain the scan number and retention time

%Add these values in fifth and sixth respectively

%end

%Find the rows with no signal information

%Generate an intensity threshold and filter signals by intensity

%Count the signals with charge 0 and display

%Count the signals with charge 1 and display

%Count the signals with other charges and display

%Show a piechar if user select

%end

[result] = charge_exploration_diferences(file, filtered)

%Obtain the number of signals without threshold intensity filter

%Obtain the number of signals with threshold intensity filter

%Obtain the percentaje of signals with intensity highier of the total number of signals

%end

[result,result2,rts] = charge_selection(fichero)

%Import sample following the instructions provided in function input description

%Obtain the rows with the first column equal to 0 and generate a matrix with this rows

%For each row of this new matrix starting from the second row

%Obtain the retention time

%end

%Delete rows with first column equal to 0 and obtain the number of rows

%For each row

%Add the scan number

%end

%Obtain the signals with charge 0

%Ask for eliminating charge equal to 1 and eliminate if user select

%Delete the selected signals

%Ask for the number of retention times of the output

%Transform the matrix in a cell array enclosing in each row the m/z and intensity values of each scan
